# Influence of Bioaerosol Source Location and Ceiling Fan Direction on Eggcrate Upper-room Ultraviolet Germicidal Irradiation

**DOI:** 10.9734/BJAST/2014/11762

**Published:** 2014-07-20

**Authors:** Sumayah F. Rahman, Stephen N. Rudnick, Sonya P. Milonova, James J. McDevitt, Edward A. Nardell

**Affiliations:** 1 Department of Environmental Health, Harvard School of Public Health, Boston, MA, USA; 2 Department of Medicine, Division of Global Health Equity, Brigham and Women’s Hospital, Boston, MA, USA; 3 Department of Global Health and Social Medicine Harvard Medical School, Boston, MA, USA

**Keywords:** Airborne disease transmission, upper-room ultraviolet germicidal irradiation, infection, bioaerosol, air disinfection, whole ceiling ultraviolet germicidal irradiation system, eggcrate, air mixing

## Abstract

**Background:**

Eggcrate upper-room ultraviolet germicidal irradiation (UVGI), an engineering control method for reducing the airborne transmission of infectious diseases, was recently developed as an alternative to conventional upper-room UVGI using conventional louvered fixtures. A UV screen, which is composed of open-cell eggcrate panels supported in a frame designed for a conventional suspended ceiling, was used to minimize UV radiation in the lower room. A ceiling fan, which was blowing upward directly above the microbiological source, provided vertical air exchange between the upper and lower room. This system has been shown to be significantly more effective than conventional upper-room UVGI.

**Study Design:**

In the present study, the microbiological source location and the airflow direction due to the ceiling fan were varied in order to evaluate their impact on germicidal efficacy.

**Results:**

The test results clearly showed that placing an aerosol source directly underneath an upward blowing ceiling fan produces the maximum efficacy.

**Conclusions:**

The likely explanation for this outcome is that the fan sucks the microorganisms emitted by the source into the UV beam before being mixed with the air in the room. This is somewhat analogous to local exhaust ventilation in which the contaminant is removed prior to being mixed with the air in the room. Thus, when possible, the ceiling fan should be blowing upward and directly above the source. However, for experimental testing, the source location should be varied in order to access the range of germicidal efficacies that can be expected.

## 1. INTRODUCTION

Upper-room ultraviolet germicidal irradiation (UVGI), an engineering control method that uses 254-nm wavelength ultraviolet light to inactivate microorganisms, reduces the airborne transmission of infectious diseases such as tuberculosis [1]. UV radiation must be confined to the upper room because it is irritating to the eyes and skin of room occupants [2]. To this end, upper-room UV fixtures have tightly spaced louvers that horizontally collimate the UV beam [2] in order to minimize UV radiation in the lower room. Unfortunately, these louvers greatly reduce the UV emission rate from the fixture [3].

Recently, Linnes et al. [4] tested an alternative concept using two unlouvered UV fixtures having a 25-W “bare” lamp, a 2.4-m high UV screen composed of open-cell eggcrate panels supported in a frame designed for a conventional suspended ceiling, and an 1.3-m upward-blowing ceiling fan hung from the center of the ceiling above the eggcrate panels. This eggcrate UVGI system maximizes the UV emission into the upper room while preventing harmful UV levels in the occupied lower room. They were able to inactivate 82% of airborne *Bacillus atrophaeus* spores in a 42-m^3^ test room, a marked improvement over the 37% inactivation they obtained with two state-of-art, conventional, commercially available, louvered UV fixtures (Hygeaire model LIND 24-EVO UV, Atlantic Ultraviolet Corp., Hauppauge, NY). Each of these fixtures contained a 25-W lamp and ballast identical to what had been used in the eggcrate UVGI system. In these tests, bacterial spores were released in the center of the room directly beneath the upward-blowing ceiling fan, whereas in real-world settings, infectious particles would usually be emitted elsewhere in the room. In this configuration, it is likely that the majority of airborne bacterial spores were immediately drawn into the irradiation zone by the fan prior to being mixed with the air in the room. If this was true, the inactivation due to UVGI may have been significantly higher than it might be for other source locations or for a downward blowing fan. In the present study, we tested this hypothesis.

## 2. METHODOLOGY

**Fig. 1:**
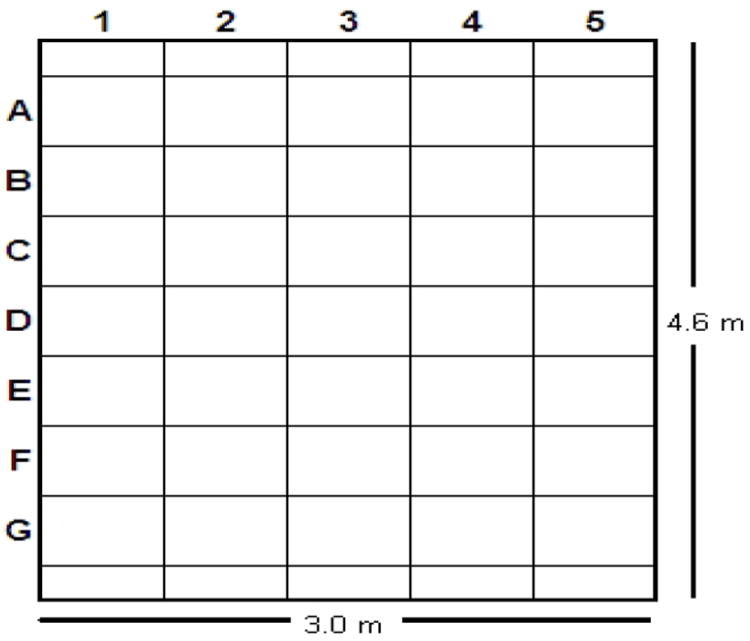
Top view of schematic diagram showing the locations of 0.61-m by 0.61-m eggcrate panels in a test room with a 3.0-m high ceiling

The apparatus and testing methodology used in the present study were identical to that of Linnes et al. [4] with the following three exceptions:

1. In the Linnes et al. [4] study, the 1.3-m, five-bladed, ceiling fan (model 28415, Hunter Fan Company, Memphis, TN) was always blowing upward at its highest speed (176 rpm), whereas in the present study the same ceiling fan was blowing at its highest speed in the same direction or downward.
2. In the Linnes et al. [4] study, the bacterial spores were released at a height of 1.5m in the center of the room, whereas in the present study, they were released either at the same location or in the corner, 280mm from the longer wall and 580mm from the shorter wall (square G1 in Fig. 1), and at a height of 1.2m.
3. In the Linnes et al. [4] study, the 0.61-m by 0.61-m eggcrate panels had 7.75-mm long vertical flow channels with a 14.6-mm by 14.6-mm cross section (Thincell TC Economy eggcrate panels, SLP Lighting, Fenton, MO). Two UV fixtures mounted on 0.61-m by 0.61-m aluminum plates were placed at locations B2 and F4 in Fig. 1 in place of eggcrate panels.

In the present study, two different types of eggcrate panels were used: a) either the same eggcrate panels used in the Linnes et al. [4] study or b) 0.61-m by 0.61-m eggcrate panels having 15.2-mm long flow channels with a 7.7-mm by 7.7-mm cross section and oriented at a 45° angle from the vertical direction (Eggcrate Core, American Louver Skokie, Illinois). When eggcrate panels with 45° angled flow channels were used, the two UV fixtures mounted on 0.61-m by 0.61-m aluminum plates were placed at locations D2 and D4 in Fig. 1 in place of eggcrate panels, rather than their previous locations of B2 and F4.

In both studies, a suspension of *Bacillus atrophaeus* spores were aerosolized in a six-jet Collison nebulizer. After steady-state conditions had been reached, samples from the room’s exhaust airflow were collected on a single-stage viable Andersen impactor with the UV fixtures turned either on or off, followed by incubation and counting of colonies.

## 3. RESULTS AND DISCUSSION

**Fig. 2:**
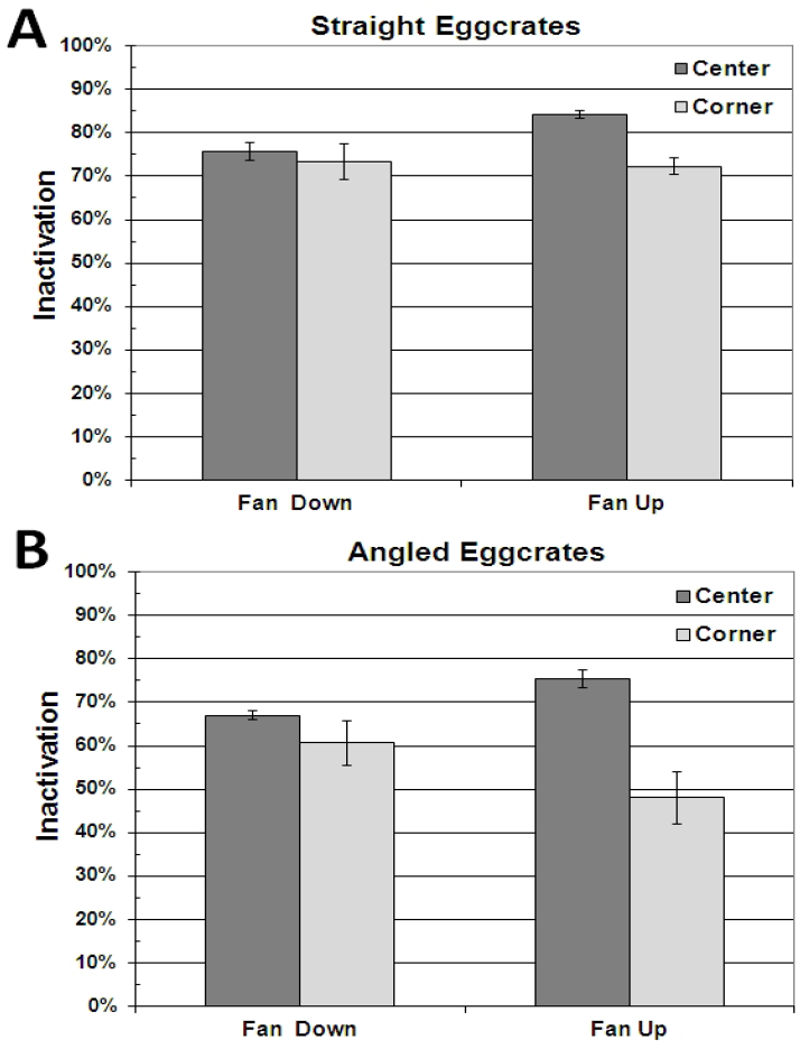
Bioaerosol tests using eggcrate panels having vertical flow channels, i.e. “straight eggcrates” (A) and eggcrate panels having 45° angled flow channels, i.e. “angled eggcrates” (B). Error bars indicate standard error of the mean for independent tests

In the present study, as shown in Fig. 2A, 84% of *Bacillus atrophaeus* spores were inactivated using eggcrate UVGI when the source was directly beneath an upward blowing ceiling fan, in good agreement with the 82% inactivation measured by Linnes et al. [4] whereas 72% were inactivated when the fan settings were left unchanged, but the source was moved to the corner of the room. The greater inactivation when the source was in the room’s center compared with the corner is statistically significant at 95% confidence (P=0.01). When the ceiling fan was blowing downward and the source was either in the center or corner of the room, 72% and 73% inactivation were measured, respectively. The difference is not statistically significant at 95% confidence (P=0.67). These results suggest that there is an advantage having the bioaerosol source directly below an upward blowing ceiling.

To confirm these findings, these tests were repeated with eggcrate panels having 45° angled flow channels. Because the angled flow channels have a smaller cross-sectional area and a longer length than the panels with vertical flow channels, airflow resistance was greater, making it more difficult for the air to flow between the upper and lower room. This additional airflow resistance resulted in less inactivation, as apparent in a comparison of Fig.2A and 2B. Nevertheless, as shown in Fig.2B, the same pattern remained. When the fan was hung from the center of the ceiling and blowing upward, 75% of bacterial spores were inactivated with the source in the center of the room compared to 48% with the source in the corner of the room, a difference that is statistically significant at 95% confidence (P=0.02). When the ceiling fan was blowing downward, there was 67% inactivation with the source in the center of the room directly below the fan compared to 61% inactivation with the source in the corner of the room. This difference was not statistically significant at 95% confidence (P=0.32). These results again support the hypothesis that having the bioaerosol source directly beneath an upward blowing ceiling fan results in greater inactivation than any of the other scenarios evaluated.

## 4. CONCLUSION

The likely explanation for the UVGI efficacy being highest when the source was directly beneath an upward blowing ceiling fan is that the fan pulls the airborne bacterial spores immediately into the UV beam prior to being mixed with the air in the room. This is somewhat analogous to local exhaust ventilation in which the contaminant is removed prior to being mixed with the air in the room. When the source is in the corner of the room and the fan is blowing upward or downward, the bacterial spores become mixed with the air in the room before reaching the UV beam. Likewise, when the source is in the center of the room and the fan is blowing downward, the bacterial spores will likely be mixed with room air prior to reaching the UV beam. Only when the source was directly beneath an upward blowing fan could the bacterial spores enter the UV beam with minimal prior mixing of the spores and room air. Because tests conducted by Linnes et al. [4] were all done with the source directly beneath an upward blowing ceiling fan, their results do not have general applicability. They apply only for very specific scenarios, such as when a bed-ridden infectious patient is directly below an upward blowing ceiling fan. For other scenarios, the percentage reduction would be expected to be less than reported by Linnes et al. [4]. Nevertheless, the use of eggcrate panels as a UV screen in place of louvers on the UV fixtures results in a marked improvement in the efficacy of upper-room UVGI compared with commercially available louvered UV fixtures as reported by Linnes et al. [4].
